# FACE-Q for Measuring Patient-reported Outcomes after Facial Skin Cancer Surgery: Cross-cultural Validation

**DOI:** 10.1097/GOX.0000000000005771

**Published:** 2024-04-29

**Authors:** Maarten J. Ottenhof, Thomas D. Dobbs, Inge Veldhuizen, Conrad J. Harrison, Michelle Marges, Erica H. Lee, Maarten M. Hoogbergen, René R.W.J. van der Hulst, Andrea L. Pusic, Chris J. Sidey-Gibbons

**Affiliations:** From * Patient-Reported Outcomes, Value & Experience (PROVE) Center, Department of Surgery, Brigham and Women’s Hospital, Boston, Mass.; † Department of Surgery, Harvard Medical School, Boston, Mass.; ‡ Department of Plastic and Reconstructive Surgery, Catharina Ziekenhuis, Eindhoven, the Netherlands; § Reconstructive Surgery and Regenerative Medicine Research Group, Institute of Life Sciences, Swansea University Medical School, Swansea, United Kingdom; ¶ Nuffield Department of Orthopaedics, Rheumatology and Musculoskeletal Sciences, University of Oxford, Oxford, United Kingdom; ∥ Dermatology Division, Memorial Sloan Kettering Cancer Center, New York, N.Y.; ** Department of Plastic and Reconstructive Surgery, Maastricht University Medical Center, Maastricht, the Netherlands; †† GROW School for Oncology and Developmental Biology, Maastricht University Medical Center, Maastricht, the Netherlands; ‡‡ MD Anderson Center for INSPiRED Cancer Care, University of Texas MD Anderson Cancer Center, Houston, Tex.

## Abstract

**Background:**

Facial skin cancer and its surgical treatment can affect health-related quality of life. The FACE-Q Skin Cancer Module is a patient-reported outcome measure that measures different aspects of health-related quality of life and has recently been translated into Dutch. This study aimed to evaluate the performance of the translated version in a Dutch cohort using modern psychometric measurement theory (Rasch).

**Methods:**

Dutch participants with facial skin cancer were prospectively recruited and asked to complete the translated FACE-Q Skin Cancer Module. The following assumptions of the Rasch model were tested: unidimensionality, local independence, and monotonicity. Response thresholds, fit statistics, internal consistency, floor and ceiling effects, and targeting were assessed for all scales and items within the scales. Responsiveness was tested for the “cancer worry” scale.

**Results:**

In total, 259 patients completed the preoperative questionnaire and were included in the analysis. All five scales assessed showed a good or sufficient fit to the Rasch model. Unidimensionality and monotonicity were present for all scales. Some items showed a local dependency. Most of the scales demonstrate ordered item thresholds and appropriate fit statistics.

**Conclusions:**

The FACE-Q Skin Cancer Module is a well-designed patient-reported outcome measure that shows psychometric validity for the translated version in a Dutch cohort, using classical and modern test theory.

**Takeaways**

**Question:** Is the FACE-Q an effective tool for assessing quality of life dimensions in the Netherlands?

**Findings:** The FACE-Q meets rigorous criteria for a reliable instrument across diverse cultural contexts.

**Meaning:** Validated for use in the Netherlands, the FACE-Q accurately assesses various health-related quality of life aspects in patients receiving plastic surgery for facial skin cancer, making it an essential outcome measure for such interventions.

## INTRODUCTION

Skin cancer is the most common malignancy worldwide,^1–3^ with the majority occurring in sun-exposed areas such as the face. The combination of facial scarring and the fear of malignancy can lead to significant psychological distress.^4–6^ As patient-reported outcome measures (PROMs) become more commonly used alongside traditional outcomes such as morbidity and mortality, there is an increasing demand for standardized and validated PROMs to measure different aspects of health-related quality of life.^7^ This PROM was previously lacking in facial skin cancers.^8^ To fill this gap, the FACE-Q Skin Cancer Module has been developed.^9–12^

The module consists of a series of questions that ask patients about the physical, emotional, and social impacts of their skin cancer and its treatment. By collecting this information, the FACE-Q Skin Cancer Module can help provide a more comprehensive understanding of the patient experience and can be used to inform the management of patients with skin cancer. The FACE-Q Skin Cancer Module can be used in a variety of settings, including in clinical practice, research, and patient education. It can help healthcare providers to better understand the needs and concerns of patients with skin cancer and can be used to guide the development of personalized treatment plans. In research settings, the FACE-Q Skin Cancer Module can be used to assess the effectiveness of different treatments or interventions for skin cancer and to identify areas for improvement in the care of patients with skin cancer. In patient education, the FACE-Q Skin Cancer Module can be used to help patients understand the potential impacts of skin cancer and its treatment on their quality of life and to provide support and resources to help them manage these impacts.

The FACE-Q Skin Cancer Module was developed using Rasch measurement theory (RMT) and probabilistic modeling to assess the performance of the questionnaire at the level of individual items. However, to use this questionnaire in those who do not speak English and compare outcomes across countries, translation into other relevant languages and further validation are required. We translated the FACE-Q Skin Cancer into Dutch, in accordance with international guidelines.^13^ Further validation of the translated version, using RMT, is required to establish whether the translated version is an adequate reflection of the original version.^14–17^ RMT is based on the idea that the difficulty or “severity” of each item in the questionnaire can be quantified and compared with the ability or “latent trait” of the individual responding to the questionnaire. RMT is often used in the development and validation of PROMs to ensure that the instrument is measuring the intended construct in a reliable and valid manner.

In this study, we evaluated the construct validity of the Dutch version of the FACE-Q Skin Cancer Module, using RMT.

## METHODS

In total, 250 patients were prospectively recruited from the department of plastic surgery and dermatology at Catharina Hospital, Eindhoven, the Netherlands. Recruitment began in September 2017, with all consecutive patients who met the eligibility criteria included. **(See document, Supplemental Digital Content 1,** which displays the inclusion and exclusion criteria. http://links.lww.com/PRSGO/D171.)

After an explanation of the study and time to consider inclusion, written informed consent was obtained. Patients were able to withdraw consent at any point. Included patients were invited by email or letter to fill out the Dutch-translated FACE-Q Skin Cancer Module at multiple time points: 1 week preoperatively, 1 week postoperatively, and 3 months postoperatively. Demographic data were collected, including diagnosis, type of reconstruction, medical history, medication, and smoking. Data were collected using Castor EDC.^18^ Medical Ethical Board Utrecht (MEC-U) approved the study (approval number: niet-WMO2017-24). Consent was obtained in writing; data were analyzed anonymously.

### Statistical Methods

Psychometric validation was performed in accordance with the guidelines of the Dutch/Flemish Patient-Reported Outcomes Measurement Information System (PROMIS) group, a pioneering collective dedicated to refining how patient well-being is quantified.^16,19^ We performed a combination of RMT and elements of classical test theory. A sample size greater than 250 was set because sample sizes between 150 and 250 provide relatively stable item calibrations or person measures.^20,21^ Missing data were managed by list-wise exclusion.

Statistical analysis was performed with R (v. 4.0.1), using the following packages: foreign (v0.8-79), dplyr (v0.8.5), mirt (v 1.32.1), eRm (v 1.0-1), ltm (v1.1-1), lordif (v.0.3-3), and WrightMap (v1.2).

### Modern Test Theory Analysis

#### Fit Statistics

Confirmatory factor analysis (CFA) was conducted to assess the unidimensionality of responses. Multiple chi-square tests were used to assess whether the data fit the Rasch model^19^ and if nonsignificant (*P* > 0.01), the data suggested a good fit. Specifically, a root mean square error of approximation (RMSEA) less than  0.06, standard root mean square residual less than 0.08, and comparative fit index (CFI) and Tucker-Lewis Index (TLI) more than 0.90–0.95 suggest that the hypothesized model structure fits the data well. In addition, infit and outfit mean-square statistics for each item were used to provide information on discrepancies in responses. Values from 0.5 to 1.5 suggest appropriate fit; values below 0.5 may indicate dependency or overfit.^22^

#### Local Dependency

Local dependency (LD) is a measure of whether a response to an item is determined solely by the respondent’s level of measured latent traits.^23^ If two items unintentionally measure an additional latent trait or when two items are too closely related, LD will exist. Yen Q3 statistic was used to examine the residual correlations between item pairs after removing the correlation accounted for by the main factor.^24^ High residual correlations (greater than 0.2 above average) were flagged and considered possible LD.

#### Response Threshold Ordering

Plotting item trace lines visualizes the probability of endorsing item response options (eg, agree and strongly agree), given a certain trait level. To determine whether response options were scored consecutively, the item response thresholds were calculated and assessed for ordering. For closely related thresholds, it would not be possible to establish true ordering with certainty.^25^ Model parameters with 95% confidence intervals are provided to better determine the stability of parameter estimates.

#### Targeting

To provide information on whether the items covered a relevant spectrum of the trait in our population, item-person plots were generated. These display the scale of the underlying trait and compare the items’ positions with the cohorts’ frequency of item endorsement. We visually examined item locations for each scale to determine if the items were evenly spread across a reasonable range that matched the range of the construct experienced by the sample. The proportion (percentage) of people responding to different items should cover the complete scale range, and in turn, the item thresholds should be spread evenly.

#### Differential Item Functioning

Differential item functioning (DIF) was examined according to age (threshold, 65 years) and sex. DIF occurs when an item functions differently for respondents with similar trait levels but different subgroups. DIF can vary depending on the level of the construct, which is called nonuniform DIF. The DIF can also be consistent across the range of constructs being measured, which is called a uniform DIF. In epidemiologic terminology, uniform DIF corresponds to confounding modification, and nonuniform DIF can be said to be an effect modification. Values (McFadden pseudo R^2^ statistic) below 0.13 are negligible, between 0.13 and 0.26, moderate; and above 0.26, large.^26^

#### Classical Test Theory

*Scaling assumptions*: To create an inter-item correlation matrix, we examined inter-item and item-scale correlations. Low or negative scores indicate translational errors.

*Internal consistency:* To assess the degree to which items on the scales were related (consistently measuring the same trait), we used Cronbach alpha. Values more than 0.70 are an acceptable minimum for group-level measurement, with values more than 0.90 suggesting item redundancy.^27^

For *responsiveness*, we computed group-level change for the “cancer worry” scale completed before and after surgery. We hypothesized that postoperative cancer worry would decrease. Preoperative and postoperative Rasch transformed scores were compared using a paired *t* test, and the effect size was calculated [ie, the mean time 1 (preoperative) minus the mean time 2 (postoperative) divided by the SD at time 1]. Cohen criteria were used to interpret the results (0.2 is small, 0.5 is moderate, and 0.8 is large).^28^

## RESULTS

### Demographic Data

A total of 287 patients were enrolled, with 28 excluded due to noncompletion of the preoperative questionnaire, leaving an analyzable dataset of 259 patients. The demographic details are presented in Table 1.

**Table 1.** Patient Demographics and Characteristics of Those Enrolled in the Dutch Cohort

Variable	All Patients (n = 259)
Age	
Mean age (SD)	69.8 (13.4)
Sex	
Male	134 (51.7%)
Female	125 (48.3%)
Comorbidities	
Cardiovascular	52 (20%)
Diabetes	19 (7.3%)
Other skin disease	58 (22.4%)
None	181 (69.9%)
Medication	
Aspirin	29 (11.2%)
Clopidogrel	6 (2.3%)
Other anticoagulation	8 (3.1%)
Immunosuppression	12 (4.6%)
Other	116 (44.8%)
None	88 (34.0%)
Histology	
BCC	259 (100%)
Previous facial surgery	
Yes	128 (49.4%
No	131 (50.6%)
Previous skin cancer	
Yes	111 (42.9%)
No	146 (56.4%)

### Modern Test Theory Analysis

Table 2 presents the results. When exploring the assumptions, all scales showed sufficient unidimensionality, and the scalability coefficients were Loevingers H more than 0.3. The two scales showed some LD between items: satisfaction with facial appearance and worry about cancer.

**Table 2.** Results of Rasch Analysis for Each Scale for the Dutch FACE-Q Skin Cancer

Subscale	Analyzable Scales	Response Thresholds	Targeting	Fit Indices Interpretation*	LD	Cronbach Alpha
Satisfaction with facial appearance	565	Ordered; item 2 close thresholds	Small ceiling effect	Sufficient fit	LD of six pairs	0.951
Appraisal of scars	302	Ordered	Large ceiling effect	Good fit	No	0.947
Cancer worry	371	Ordered; item 10 close thresholds	Small floor effect Coverage good for first five items	Good fit	LD of three pairs	0.904
Satisfaction with information: appearance	489	Ordered	Good coverage with small ceiling effect	Sufficient fit	No	0.943
Appearance-related psychosocial distress	277	Ordered; items 2, 5, 8 close thresholds	Large floor effect	Good fit	No	0.885

* Data are displayed in Supplemental Digital Contents 2–5 and 10.

Supplemental Digital Content 2 shows the results of the CFA. (**See table 1, Supplemental Digital Content 2,** which displays the fit statistics. http://links.lww.com/PRSGO/D172.) The CFA was performed, the combined timepoints producing the highest fit indices. For the cancer worry scale, CFI = 0.96, TLI = 0.96, SRMR = 0.095, and RMSEA = 0.036. For satisfaction with facial appearance, CFI = 0.968, TLI = 0.67, SRMR = 0.152, and RMSEA = 0.066. For appearance-related psychosocial distress, CFI = 0.976, TLI = 0.98, SRMR = 0.086, RMSEA = 0.095. For satisfaction with appearance, information CFI = 0.967, TLI = 0.98, SRMR = 0.156, RMSEA = 0.106. For appraisal of scars, CFI = 0.957, TLI = 0.96, SRMR = 0.0866, and RMSEA = 0.056. Thresholds were mostly successively ordered, although some items had disordered thresholds or were closely related so that true ordering could not be established (displayed in Supplemental Digital Content 3): item 1 and 2 from facial appearance; items 2, 5, and 8 from appearance-related psychosocial distress; and item 10 in cancer worry. [**See graph, Supplemental Digital Content 3,** which displays the item-person plot. The item-person plot displays the distribution of responses for both individuals and test items. It provides insights into the effectiveness of the scale and the quality of the items by showing the response option distribution for each person (on the left) and each item (on the right). http://links.lww.com/PRSGO/D173.] Supplemental Digital Content 4 shows the model parameters with 95% confidence intervals. [**See table 2, Supplemental Digital Content 4,** which displays the model parameters. The thresholds for each item on the scales are listed with a 95% confidence interval using the Rasch model for estimation. The Rasch model was used to estimate the thresholds for each item on the scales and the corresponding 95% confidence intervals. The thresholds represent the estimated difficulty of each item (and its response options), and the confidence intervals provide a measure of uncertainty around the estimates. http://links.lww.com/PRSGO/D174.]

The item-person plots (displayed in **Supplemental Digital Content 3,** http://links.lww.com/PRSGO/D173) that showed ceiling effects were the facial appearance and appraisal of scars scales. The IPPs for cancer worry and appearance-related psychosocial distress showed floor effects. The infit and outfit statistics are shown in Supplemental Digital Content 5. [**See graph, Supplemental Digital Content 5,** which displays the infit and outfit statistics. Infit is more sensitive to unexpected patterns of observation by people on items that are roughly targeted to them (the degree to which the observed responses for an item conform to the predictions of the model). Outfit is more sensitive to unexpected observations by people regarding items that are relatively easy or difficult for them (the degree to which the observed responses for an item deviate from the predictions of the model). Acknowledging this measure is not perfect; values between 0.5 and 1.5 are considered to be productive for measurement. http://links.lww.com/PRSGO/D175.]

They represent the differences between the observations and their expected values, according to the Rasch model. Infit is more sensitive to the pattern of responses to the items targeted by an individual. Outfit was more sensitive to responses to items with difficulty far from the person. A few items showed values outside the normal range, and seven items showed a slight overfit. We looked for DIF across all scales. No items or scales were flagged for DIF for grouped age (threshold 65 years) and sex (data shown in Supplemental Digital Content 6). [**See graph, Supplemental Digital Content 6,** which displays the differential item functioning (DIF). DIF analysis is used to evaluate whether an item, on a scale, functions differently for different groups of respondents. Grouped age (threshold 65) and gender were checked for DIF: McFadden pseudo R^2^ statistic: each item below 0.13 is negligible, below 0.13 and 0.26, moderate, and between 0.26 is large. The results of the DIF analysis can be used to identify items that may be biased or functioning differently for different groups of respondents. http://links.lww.com/PRSGO/D176.] Supplemental Digital Contents 7–9 show raw scores from FACE-Q scales (Table 2). (**See table, Supplemental Digital Content 7**, which displays the raw scores for cancer worry, satisfaction with facial appearance, appearance-related psychosocial distress, satisfaction with appearance information, and appraisal of scars scales recorded on the FACE-Q. Different timepoints in columns and scores were recorded on a Likert-type scale of 1–4. http://links.lww.com/PRSGO/D177.) (**See table, Supplemental Digital Content 8,** which displays the raw scores for cancer worry, satisfaction with facial appearance, appearance-related psychosocial distress, satisfaction with appearance information, and appraisal of scars scales recorded on the FACE-Q. Different timepoints in columns and scores were recorded on a Likert-type scale of 1–4. http://links.lww.com/PRSGO/D178.) (**See table, Supplemental Digital Content 9,** which displays the raw scores for cancer worry, satisfaction with facial appearance, appearance-related psychosocial distress, satisfaction with appearance information, and appraisal of scar scales recorded on the FACE-Q. Different timepoints in columns and scores were recorded on a Likert-type scale of 1–4. http://links.lww.com/PRSGO/D179.) [**See table, Supplemental Digital Content 10,** which displays the item characteristic curves. Item characteristic curves are graphical representations of the relationship between a latent trait on the *x*-axis (as measured by the FACE-Q scales) and the probability of endorsing each response option across latent trait levels on the *y*-axis. The position of the curve reflects the difficulty of the test item and the discriminating area of the item (that is, its ability to differentiate between individuals with different levels of the latent trait), http://links.lww.com/PRSGO/D180.]

### Classical Test Theory

Inter-item correlations did not reveal any negatively scored items; therefore, rescoring was not required. Cronbach alpha was more than 0.7 for all scales, with some demonstrating values more than 0.95 (Table 2).

Responsiveness: Seventy-nine respondents completed the cancer worry scale before and after the surgery. A significant decrease in mean scores was observed between the preoperative score [35.2 (SD 19.1)] and postoperative score [28.3 (SD 21.1)] on the cancer worry scale (*P* < 0.001, effect size 0.38).

## DISCUSSION

This study evaluated the performance of the Dutch FACE-Q Skin Cancer Modules to determine whether they fit the Rasch model and provide response-option–level measurements in the Dutch population. Overall, psychometric analysis provided evidence of an acceptable Rasch model fit for the analyzed scales. However, a perfect fit was not observed. Recognizing that universally accepted fit statistics do not exist, model fit was assessed using several indices. When the model assumptions are supported by the data, strict adherence to the model fit is not vital, given the limits of the acceptable fit indices.^19^ The issue is the degree to which misfit affects the valid scaling of individual differences. When confirming the validity of an existing questionnaire, such as the FACE-Q Skin Cancer Module, a certain level of misfit is acceptable, and several reasons could explain this, such as subtle violations of the model assumptions. For example, sample size and mild nonnormality distribution (in our sample due to kurtosis) could have hampered the precise use of fit indices because they are chi-square-based.^19,29^ Additionally, it can indicate minor violations of unidimensionality. However, research suggests that IRT models are relatively robust to minor violations of unidimensionality, especially when the factors are highly correlated.^30^

A degree of LD was observed in the satisfaction with facial appearance and cancer worry scales. The former also had a Cronbach alpha more than 0.95, which may suggest item redundancy, and Cronbach alpha loses its interpretation as a lower bound of reliability.^31^ Redundant items were removed. In addition, thresholds should be interpreted cautiously in the presence of LD. The thresholds may be closely related or reversed. Another solution to locally dependent items (assuming unidimensionality) could be the utilization of “computerized adaptive testing”, which skips redundant items when they are mistargeted by the individual. The computerized adaptive testing algorithm has already been established to be feasible for the FACE-Q.^32^

In this sample, items 2 (satisfaction with facial appearance) and 10 (cancer worry) had closely situated and potentially disordered thresholds. This may occur when respondents have difficulty distinguishing between two response options (eg, “very dissatisfied” and “dissatisfied”) in the context of a particular item; therefore, successive response options do not reflect an increase in the underlying trait. During development, this can be resolved by collapsing the two response options. However, before collapsing the response options, altering the scale would hamper the international use and comparison of the FACE-Q results. If disordered thresholds are not observed in samples from other countries, future research could aim to determine whether items demonstrate different item functioning by country. We found no DIF for grouped age (threshold 65 years) and sex in the Dutch data. This indicated that the items did not cause confounding or effect modifications.

Only the “cancer worry” scale was tested for responsiveness to change because it was the only scale in which an expected change in postoperative scores could be assumed. For instance, some respondents may experience improvement upon removal of their skin cancer, whereas others may feel that reconstruction of the resulting defect with a local flap is worse.

A limitation of this study was that the patients included in this cohort were provided with an electronic form that contained errors. The first item of appearance-related psychosocial distress, “I feel self-aware of my…,” became “I feel aware of my….” This resulted in the exclusion of responses to items in the analysis. We treated repeated PROM measurements independently to include a broad range of item responses in our sample and leverage all available data. However, this approach may have inflated fit statistics, reduced the size of confidence intervals, and introduced bias if respondents interpreted and responded to the PROMs differently than the broader population. We still considered this approach preferable to using baseline-only data in this study.

The Rasch model fit was sufficient, and the parameter estimates were relatively stable. However, a larger sample size would allow our claim of fit to the Rasch model parameters to be made with even more confidence.

Future analyses using larger datasets might strengthen these estimates. Continuing routine data collection using the Dutch FACE-Q Skin Cancer Module should be made using Dutch translation to focus on subgroup analysis. The translated version of the FACE-Q Skin Cancer Module is a valid and reliable tool for measuring health-related quality of life in Dutch patients with facial skin cancer. The module demonstrates good psychometric properties and can be used by healthcare professionals and researchers to assess the impact of skin cancer and its treatment on patients’ quality of life. This information can be used to improve the care and management of patients with skin cancer.

## CONCLUSIONS

This is the largest validation study of the FACE-Q Skin Cancer Module, and supports the instrument’s construct validity in the Netherlands.

## DISCLOSURES

Andrea Pusic is codeveloper of the FACE-Q and receives royalties when it is used in for-profit, industry-sponsored clinical trials. The FACE-Q can be used free of charge for nonprofit purposes (eg, clinicians, researchers, and students). All the other authors have no financial interest to declare in relation to the content of this article. The FACE-Q is owned by Memorial Sloan-Kettering Cancer Center and McMaster University. Conrad J. Harrison is funded by a National Institute for Health Research (NIHR) Doctoral Research Fellowship (NIHR300684). The views expressed are those of the authors and not necessarily those of the National Health Service (NHS), the NIHR, or the Department of Health and Social Care.

## ACKNOWLEDGMENT

We thank our colleagues at the PROVE Center for their technical assistance and insight.

## Supplementary Material


