# Supplementary material for: FACE-Q for Measuring Patient-reported Outcomes after Facial Skin Cancer Surgery: Cross-cultural Validation
Source: Plast Reconstr Surg Glob Open. 2024 Apr 29;12(4):e5771. doi: 10.1097/GOX.0000000000005771 (PMC11057807; doi:10.1097/GOX.0000000000005771)
Supplement: Supplementary file 1 [file gox-12-e5771-s001.pdf]

SDC 1 – Inclusion and exclusion criteria.

Inclusion criteria

- Active treatment of Non-melanoma skin cancer of the face
- Over 18 years of age
- Willing to participate

Exclusion criteria

- Inability to consent to participation in the study
- Known learning difficulties or conditions such as dementia, depression, psychosis
- Insufficiency in Dutch
- Non-excisional treatment such as chemotherapy or laser
- Free tissue reconstruction
