# Supplementary material for: FACE-Q for Measuring Patient-reported Outcomes after Facial Skin Cancer Surgery: Cross-cultural Validation
Source: Plast Reconstr Surg Glob Open. 2024 Apr 29;12(4):e5771. doi: 10.1097/GOX.0000000000005771 (PMC11057807; doi:10.1097/GOX.0000000000005771)
Supplement: Supplementary file 2 [file gox-12-e5771-s002.pdf]

SDC 2 – Fit statistics

Fit indices for scales					
		Confirmatory Factor Analasys (CFA)			
Scale	Samples time	CFI[1]	TLI[2]	SRMR[3]	RMSEA[4]
Reference value		>0.90	>0.95	<0.08	< 0.06
Cancer worry	Pre-op	0.804	0.748	0.094	0.178
	3 m	0.73	0.653	0.141	0.219
	Combined	0.96	0.96	0.095	0.036
Satisfaction with facial appearance	Pre-op	0.782	0.709	0.088	0.23
	1 w	0.832	0.775	0.085	0.24
	3 m	0.678	0.57	0.095	0.369
	Combined	0.968	0.67	0.152	0.066
Appearance-related psychosocial distress	Pre-op	0.948	0.922	0.058	0.119
	1 w	0.977	0.965	0.027	0.111
	3 m	0.981	0.971	0.026	0.095
	Combined	0.976	0.98	0.086	0.11
Satisfaction with appearance information	Pre-op	0.92	0.867	0.044	0.204
	1 w	0.929	0.881	0.046	0.207
	3 m	0.967	0.945	0.04	0.124
	Combined	0.844	0.84	0.156	0.106
Appraisal of scars	1 w	0.962	0.946	0.034	0.116
	3 m	0.936	0.911	0.05	0.14
	Combined	0.957	0.96	0.0866	0.056
