# Supplementary figures and images for: FACE-Q for Measuring Patient-reported Outcomes after Facial Skin Cancer Surgery: Cross-cultural Validation
Source: Plast Reconstr Surg Glob Open. 2024 Apr 29;12(4):e5771. doi: 10.1097/GOX.0000000000005771 (PMC11057807; doi:10.1097/GOX.0000000000005771)

**SDC 3 - Item Person Plots for the FACE-Q Skin Cancer module scales.**

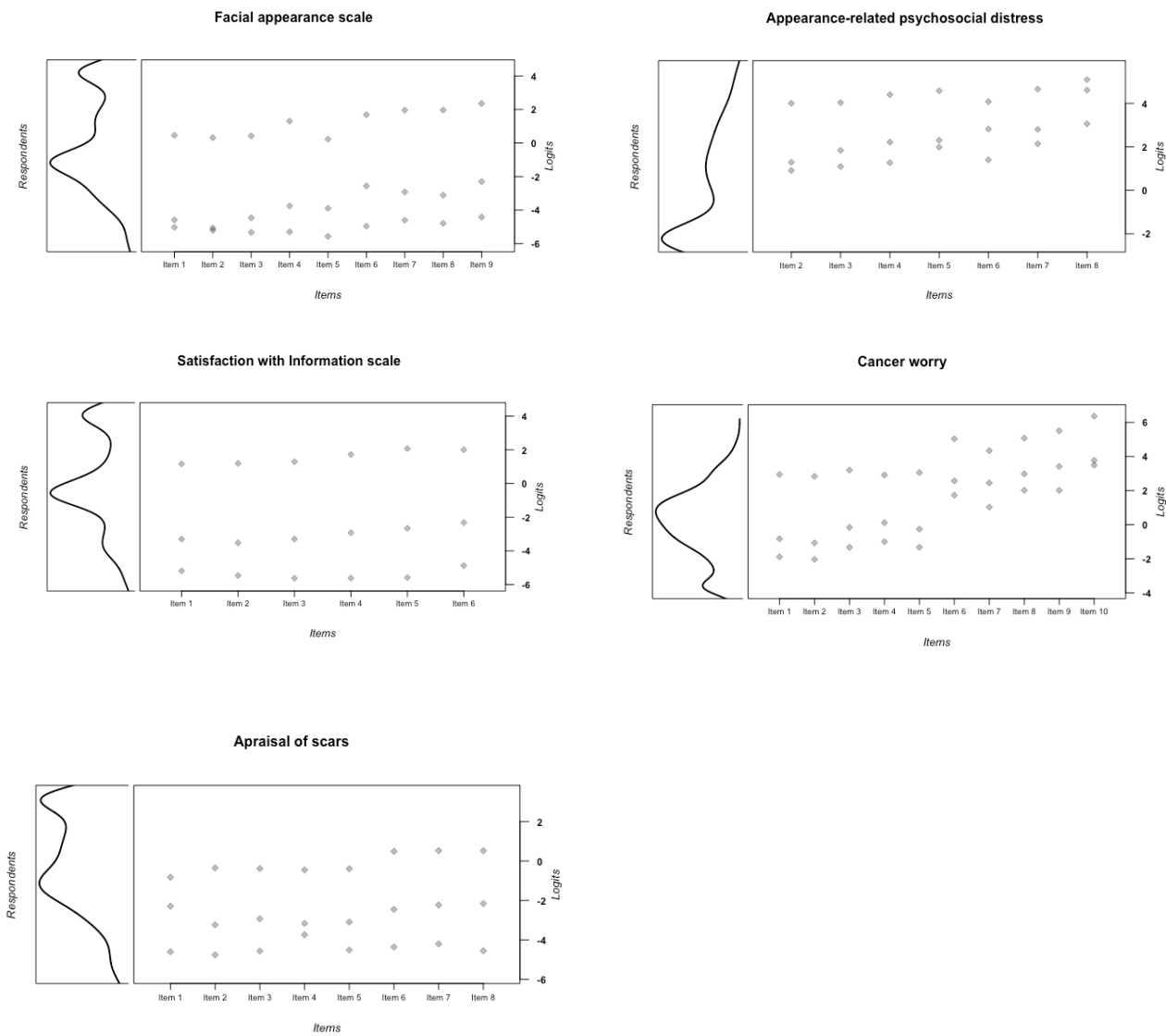

Supplement: Supplementary file 3 [file gox-12-e5771-s003.pdf]
