# Supplementary material for: FACE-Q for Measuring Patient-reported Outcomes after Facial Skin Cancer Surgery: Cross-cultural Validation
Source: Plast Reconstr Surg Glob Open. 2024 Apr 29;12(4):e5771. doi: 10.1097/GOX.0000000000005771 (PMC11057807; doi:10.1097/GOX.0000000000005771)
Supplement: Supplementary file 5 [file gox-12-e5771-s005.pdf]

SDC 4 – Infit and Outfit statistics for the FACE-Q Skin Cancer scales.

Subscale	**Item**	**Infit**	**Outfit**
Satisfaction with facial appearance	1	0.797	0.999
	2	0.549	0.741
	3	0.565	0.698
	4	0.533	0.684
	5	0.742	0.801
	6	0.751	0.857
	7	0.653	0.786
	8	0.543	0.663
	9	0.621	0.661
Appraisal of scars	1	1.167	1.261
	2	0.710	1.011
	3	0.521	0.655
	4	0.649	0.809
	5	0.448	0.600
	6	0.676	0.819
	7	0.493	0.582
	8	0.703	0.816

Cancer worry	1	0.797	0.795
	2	0.793	0.814
	3	0.628	0.682
	4	0.907	0.924
	5	0.780	0.820
	6	0.640	0.881
	7	0.653	0.761
	8	1.893	0.927
	9	0.968	1.399
	10	0.914	1.412
Satisfaction with appearance information	1	0.831	1.036
	2	0.523	0.654
	3	0.483	0.605
	4	0.484	0.587
	5	0.435	0.517
	6	0.702	0.787
Appearance-related psychosocial distress	2	0.588	0.916
	3	0.611	0.989
	4	0.622	1.112
	5	0.231	0.484
	6	0.323	0.497
	7	0.424	0.651
	8	0.873	1.203
