# Supplementary material for: FACE-Q for Measuring Patient-reported Outcomes after Facial Skin Cancer Surgery: Cross-cultural Validation
Source: Plast Reconstr Surg Glob Open. 2024 Apr 29;12(4):e5771. doi: 10.1097/GOX.0000000000005771 (PMC11057807; doi:10.1097/GOX.0000000000005771)
Supplement: Supplementary file 6 [file gox-12-e5771-s006.pdf]

Scale	Items	DIF Age and Gender		
		Uniform (R1,2)	In General (R1,3)	Non-Uniform (R2,3)
**Cancer worry**	1…skin cancer	0.0016	0.0042	0.0025
		0.0057	0.0103	0.0046
	2…may come back	0.0006	0.0006	0.0000
		0.0002	0.0003	0.0001
	3…anxious	0.0024	0.0024	0.0000
		0.0093	0.0102	0.0009
	4…shape	0.0000	0.0018	0.0018
		0.0045	0.0046	0.0002
	5…get worse	0.0004	0.0038	0.0034
		0.0013	0.0017	0.0003
	6…activities	0.0030	0.0038	0.0007
		0.0003	0.0004	0.0001
	7…worry every day	0.0011	0.0014	0.0003
		0.0006	0.0009	0.0003
	8….enjoying life	0.0017	0.0019	0.0002
		0.0001	0.0002	0.0001
	9…die	0.0003	0.0007	0.0004
		0.0089	0.0106	0.0017
	10…relationships	0.0012	0.0089	0.0077
		0.0000	0.0024	0.0024
**Satisfaction with facial appearance**	1…shape	0.0160	0.0169	0.0009
		0.0004	0.0045	0.0041
	2…contour	0.0001	0.0099	0.0098
		0.0011	0.0021	0.0008
	3…symmetrical	0.0019	0.0052	0.0033
		0.0029	0.0120	0.0130
	4…uniform	0.0001	0.0002	0.0001

		0.0135	0.0273	0.0138
	5…sides match	0.0005	0.0006	0.0011
		0.0216	0.0342	0.0126
	6…how smooth	0.0005	0.0055	0.0050
		0.0116	0.0164	0.0048
	7…in photos	0.0053	0.0086	0.0034
		0.0037	0.0111	0.0074
	8…in the mirror	0.0048	0.0051	0.0003
		0.0003	0.0003	0.0000
	9…close-up	0.0006	0.0023	0.0017
		0.0049	0.0269	0.0220
**Appearance-related psychosocial distress**	2…insecure	0.0068	0.0070	0.0002
		0.0185	0.0194	0.0009
	3…unhappy	0.0014	0.0014	0.0000
		0.0026	0.0041	0.0016
	4…anxious	0.0000	0.0050	0.0050
		0.0037	0.0069	0.0032
	5…stressed	0.0007	0.0016	0.0009
		0.0051	0.0054	0.0003
	6…embarrassed	0.0340	0.0426	0.0086
		0.0000	0.0270	0.0270
	7…depressed	0.0395	0.0523	0.0129
		0.0097	0.0313	0.0216
	8…avoid friends	0.0357	0.0368	0.0011
		0.0063	0.1257	0.1195
**Satisfaction with appearance information**	1…change	0.0018	0.0025	0.0007
		0.0024	0.0026	0.0002
	2…when healed	0.0001	0.0021	0.0020
		0.0027	0.0033	0.0005
	3…how toned	0.0005	0.0005	0.0001
		0.0000	0.0189	0.0189

	4…scars over time	0.0061	0.0063	0.0002
		0.0009	0.0038	0.0029
	5…look like	0.0005	0.0009	0.0004
		0.0000	0.0002	0.0001
	6…help with scarring	0.0085	0.0088	0.0038
		0.0001	0.0097	0.0097
**Appraisal of scars**	1…color	0.0000	0.0000	0.0000
		0.0001	0.0001	0.0001
	2…crooked	0.0067	0.0112	0.0045
		0.0236	0.0251	0.0015
	3…how wide	0.0114	0.0119	0.0005
		0.0135	0.0185	0.0050
	4…how noticeable	0.0126	0.0159	0.0033
		0.0001	0.0032	0.0031
	5…length	0.0025	0.0025	0.0001
		0.0000	0.0076	0.0076
	6…how thick	0.0000	0.0025	0.0024
		0.0027	0.0024	0.0017
	7…how noticeable	0.0007	0.0010	0.0004
		0.0038	0.0038	0.0000
	8…location	0.0006	0.0015	0.0009
		0.0007	0.0016	0.0010
