# Supplementary material for: FACE-Q for Measuring Patient-reported Outcomes after Facial Skin Cancer Surgery: Cross-cultural Validation
Source: Plast Reconstr Surg Glob Open. 2024 Apr 29;12(4):e5771. doi: 10.1097/GOX.0000000000005771 (PMC11057807; doi:10.1097/GOX.0000000000005771)
Supplement: Supplementary file 10 [file gox-12-e5771-s010.pdf]

SDC 10 - Item Characteristic Curves for FACE-Q Skin Cancer scales

Appearance-related psychological distress: 277 obs.

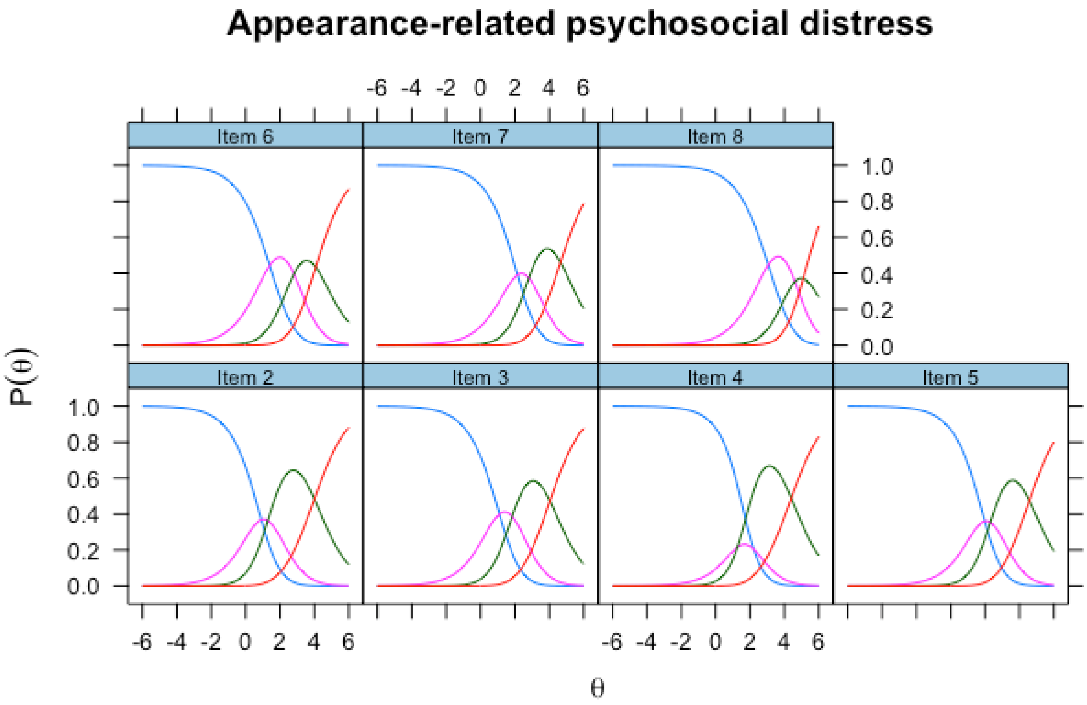

Satisfaction with information: 250 obs.

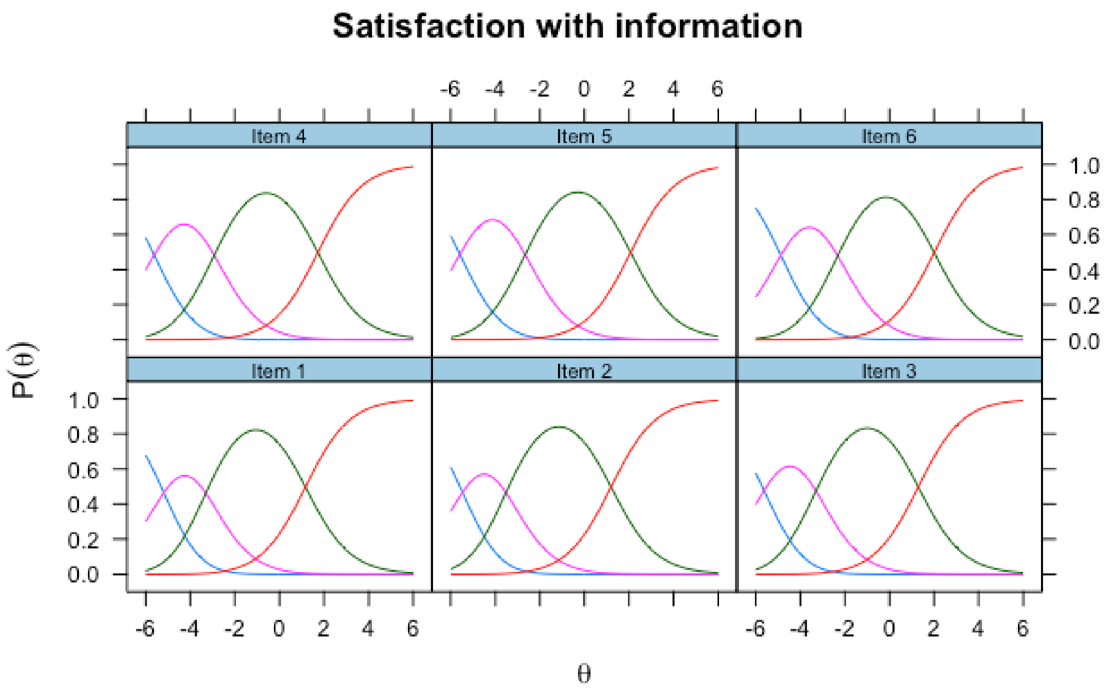

Appraisal of scars: 302 obs.

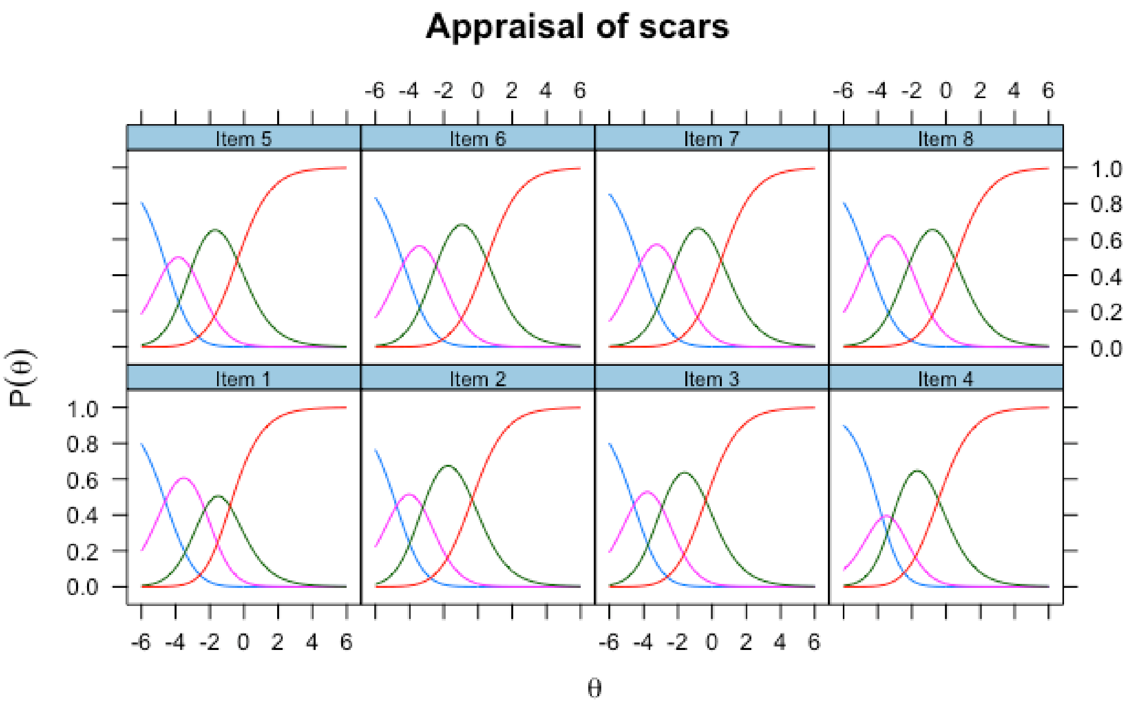

Satisfaction with facial appearance: 565 observations from combined observations.

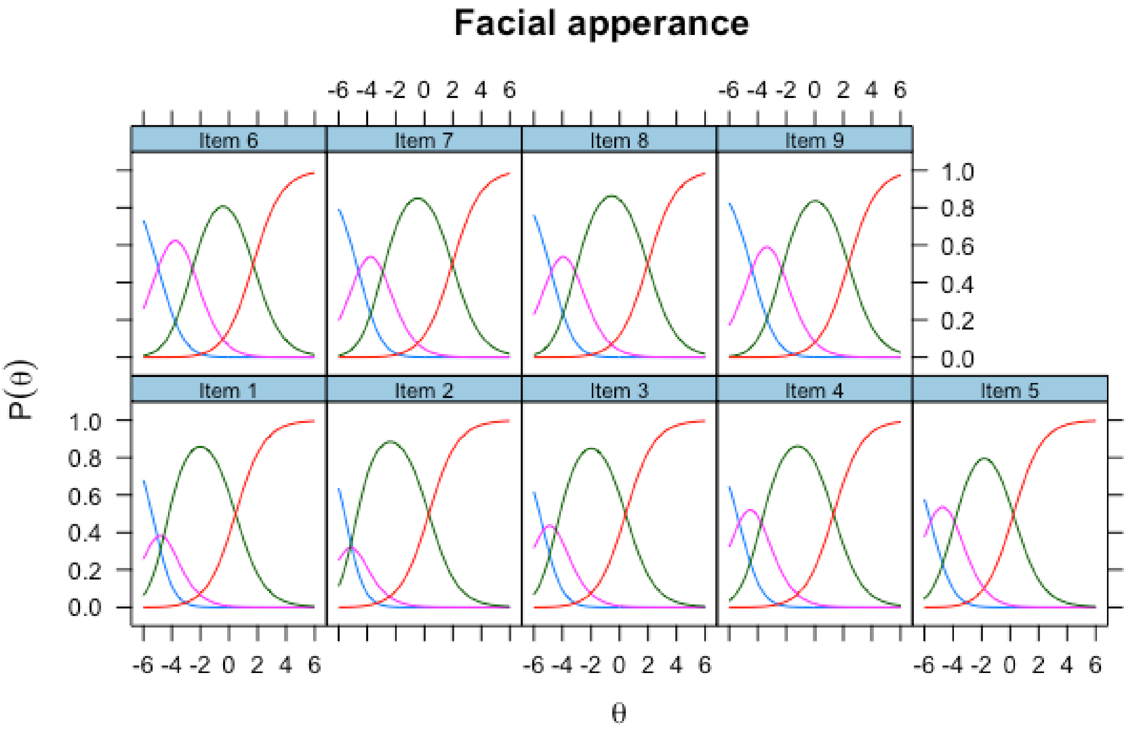

Cancer worry: 371 observations

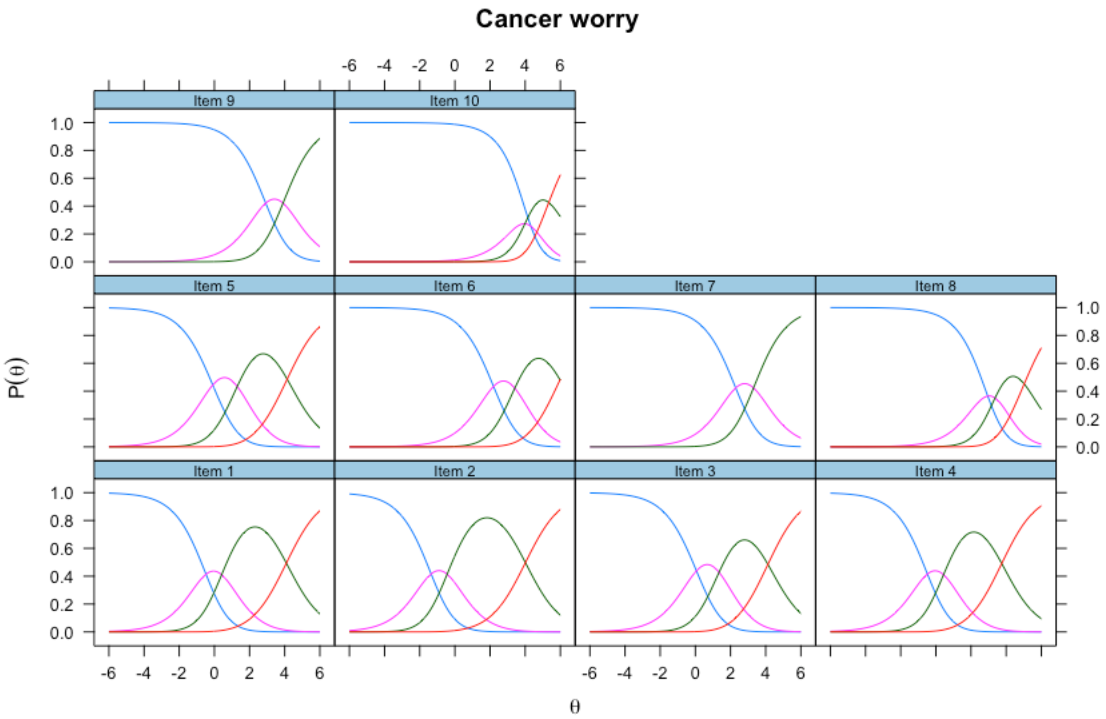

Cancer worry
Item 9
Item 10
Item 5
Item 6
Item 7
Item 8
Item 1
Item 2
Item 3
Item 4
P(θ)
θ
